# Supplementary material for: A Survey on Vaccination and Disease Occurrence in Municipal and Non-Profit Animal Shelters in Portugal
Source: Animals (Basel). 2023 Aug 27;13(17):2723. doi: 10.3390/ani13172723 (PMC10486493; doi:10.3390/ani13172723)
Supplement: Supplementary file 1 [file animals-13-02723-s001.zip › animals-2550699-supplementary.pdf]

Instructions to fill questionnaire

Subject: Request for disclosure of questionnaire

ANVETEM
https://anvetem.wordpress.com
Email: geral.anvetem@gmail.com

Dear President of ANVETEM

Within the scope of Shelter Veterinary Medicine, and with the aim to characterize the situation of animal shelters in Portugal, a questionnaire was developed by Municipal Veterinarians and Researchers from the do Instituto de Ciências Biomédicas Abel Salazar (ICBAS) of the University of Porto. In this context, we would like to request your collaboration in its disclosure by your associates.
The questionnaire must be completed by the Veterinarian responsible for the Official Animal Collection Center/Municipal Kennel, with only one response per institution. It takes approximately 30 minutes to complete and is available electronically until March 31st: https://inqueritos.up.pt/index.php?r=survey/index&sid=492317&lang=pt
Participation is voluntary and anonymous. The data collected will be treated in accordance with the Data Protection Law. The study was approved by the Ethics Committee of ICBAS, University of Porto, under number 2020/CE/P022.

Thank you in advance for your availability and collaboration in disseminating this questionnaire.

Best regards,

The Shelter Veterinary Medicine group

Adélia Alves Pereira
Alexandra Müller
Cláudia Baptista
Eduarda Gomes Neves
Pedro Osório
Sara Marques

For additional questions:
Alexandra Müller - ammuller@icbas.up.pt
Adélia Alves Pereira - adeliamedicina.abrigo@gmail.com

Questionnaire

Section A: I. GENERAL INFORMATION

A1. Are you a Veterinarian?

Yes ☐
No ☐

A2. Do you work at Association/ Animal Shelter?

Yes ☐
No ☐

A3. What type of Shelter are you responsible for?

Non-profit Shelter with Adoption ☐
Sanctuary ☐
Municipal Shelter ☐
Centro de Recolha Oficial ☐
Inter-Municipal Shelter ☐
Other

Other

A4.

According to the map indicate the location of the Shelter:

The Autonomous Regions of Madeira and Azores are included in the answers despite not being on the map.

Source of the image: Wikipédia

Non-response ☐
Minho ☐
Trás-os-Montes e Alto Douro ☐
Douro Litoral ☐
Beira Alta ☐
Beira Litoral ☐
Beira Baixa ☐
Extremadura ☐
Ribatejo ☐
Alto Alentejo ☐
Baixo Alentejo ☐
Algarve ☐
Madeira ☐
Açores ☐

A9. **The shelter is designed to which kind of animals?**

	Yes	No
Dogs	☐	☐
Cats	☐	☐
Other species	☐	☐

A10. **What is the current number (approximately) of dogs?**

A11. **What is the current number (approximately) of cats?**

Section H: VIII. ANIMAL BEHAVIOR

H10. Where animals are assisted, excluding sterilizations?

- At the facility, by the responsible veterinarian ☐
- At a Veterinary Medical Center (CAMV) ☐
- At the facility, by an external veterinarian ☐
- Other ☐

Other

Section I: IX. PERMANENCE AND ADOPTION

I1. What is the approximate percentage of animals that have been in the shelter for more than 1 year?

	< 5%	5-10%	>10-25%	>25--50%	> 50%	Non-applicable
**Dogs**	☐	☐	☐	☐	☐	☐
**Cats**	☐	☐	☐	☐	☐	☐

Section J: X. CLINICAL ASPECTS

J1. There are individual medical records?

Yes ☐
No ☐

J2. What prophylactic interventions are routinely performed?

External parasite control ☐
Internal parasite control ☐
Vaccination ☐

J3. Are there financial restrictions for regular application of these prophylactic interventions?

Yes ☐
No ☐

J4. What vaccines are available for dogs?

- Rabies ☐
- Pentavalent DHPPi+L ☐
- Kennel cough ☐
- Unavailable ☐
- Non-applicable ☐

J5. What vaccines are available for cats?

- Trivalent (RCP) ☐
- FeLV ☐
- Rabies ☐
- Unavailable ☐
- Non-applicable ☐

J6. At the moment, what is the vaccination coverage of housed animals?

	>80%	50-80 %	<50%	Non-applicable
Dogs	☐	☐	☐	☐
Cats	☐	☐	☐	☐

J7. Regarding the vaccination protocol for puppies and kittens, indicate:

- Are vaccinated repeatedly every 2-4 weeks? ☐
- The last booster of the primary vaccination is given after 16 weeks of age? ☐
- Other ☐

Other

J8. Regarding the vaccination protocol for adults, what is the periodicity of revaccination?

	Annual	Every 3 years	No revaccination	Non-applicable
DHPPi+L in dogs	☐	☐	☐	☐
RCP in cats	☐	☐	☐	☐

J9. In dogs, what is the occurrence of the following infectious diseases in the last 12 months?

In this survey an "outbreak" is considered to be 2 or more animals affected in the same period.

	No disease	1-2 cases (individual animals)	>2 cases	at least nos 1 one outbreak	un known	Non-applicable
Canine distemper	☐	☐	☐	☐	☐	☐
Parvovirus	☐	☐	☐	☐	☐	☐
Canine coronavirus	☐	☐	☐	☐	☐	☐
Kennel cough	☐	☐	☐	☐	☐	☐
Mange	☐	☐	☐	☐	☐	☐
Dermatophytosis	☐	☐	☐	☐	☐	☐

J10. What are the main diagnostic methods?

	Clinical diagnosis	Rapid test / laboratory	Necro psy	Non-applicable
Canine distemper	☐	☐	☐	☐
Parvovirus	☐	☐	☐	☐
Canine coronavirus	☐	☐	☐	☐
Kennel cough	☐	☐	☐	☐

J11. In cats, what is the occurrence of the following infectious diseases in the last 12 months?

In this survey an "outbreak" is considered to be 2 or more animals affected in the same period..

	No disease	1-2 cases (individual animals	>2 cases	at least one outbreak	Un known	Non-applicable
Feline panleukopenia	☐	☐	☐	☐	☐	☐
Cat flu	☐	☐	☐	☐	☐	☐
Feline infectious peritonitis	☐	☐	☐	☐	☐	☐
Mange	☐	☐	☐	☐	☐	☐
Dermatophytosis	☐	☐	☐	☐	☐	☐

J12. **What are the main diagnostic methods?**

	Clinical diagnosis	Rapid test/ laboratory	Necro psy	Non-applicable
Feline panleukopenia	☐	☐	☐	☐
Cat flu	☐	☐	☐	☐
Feline infectious peritonitis	☐	☐	☐	☐

J13. **In cats, rapid tests are made for retroviruses?**

- FIV ☐
- FeLV ☐
- Not performed ☐
- Non-applicable ☐

J14. **In cats, a positive rapid test result is confirmed by another rapid or laboratory test?**

- FIV ☐
- FeLV ☐
- No confirmation ☐
- Non-applicable ☐

Assunto: Pedido de divulgação de questionário

ANVETEM
https://anvetem.wordpress.com
Email: geral.anvetem@gmail.com

Ex.mo Senhor
Presidente da ANVETEM
No âmbito da Medicina Veterinária de abrigo, e com o objectivo de caracterizar a situação dos abrigos de animais em Portugal, foi desenvolvido um questionário por Médicos Veterinários Municipais e Investigadores do Instituto de Ciências Biomédicas Abel Salazar (ICBAS) da Universidade do Porto. Neste contexto, vimos solicitar a vossa colaboração na sua divulgação pelos vossos associados.
O questionário deve ser preenchido pelo Médico Veterinário responsável pelo Centro de Recolha Oficial de Animais/Canil Municipal, devendo ocorrer apenas uma resposta por instituição. O seu preenchimento demora aproximadamente 30 minutos e está disponível por via eletrónica até ao dia 31 de Março:
https://inqueritos.up.pt/index.php?r=survey/index&sid=492317&lang=pt
A participação é voluntária e anónima. Os dados recolhidos serão tratados de acordo com a Lei de Proteção de Dados. O estudo foi aprovado pela Comissão de Ética do ICBAS, Universidade do Porto, com o número 2020/CE/P022.

Agradecemos, desde já, a disponibilidade e colaboração na divulgação deste questionário.

Com os melhores cumprimentos,

O grupo de Medicina Veterinária de Abrigo
Adélia Alves Pereira
Alexandra Müller
Cláudia Baptista
Eduarda Gomes Neves
Pedro Osório
Sara Marques

Para questões adicionais:
Alexandra Müller - ammuller@icbas.up.pt
Adélia Alves Pereira - adeliamedicina.abrigo@gmail.com

Secção A: I. INFORMAÇÃO GERAL

A1. É Médico/a Veterinário?

Sim ☐
Não ☐

A2. Trabalha em Alojamento/ Abrigo Animais?

Sim ☐
Não ☐

A3. Qual o tipo de Alojamento pelo qual é responsável?

Alojamento para Hospedagem sem Fins Lucrativos - com adoção ☐
Alojamento para Hospedagem sem Fins Lucrativos - santuário ☐
Centro de Recolha Oficial Municipal ☐
Centro de Recolha Oficial Intermunicipal ☐
Outro ☐

Outro

A4.

De acordo com o mapa supra indique a localização do Abrigo:

As Regiões Autónomas da Madeira e Açores estão contempladas nas respostas apesar de não se encontrarem no mapa.

Fonte da imagem: Wikipédia

Não responde ☐
Minho ☐
Trás-os-Montes e Alto Douro ☐
Douro Litoral ☐
Beira Alta ☐
Beira Litoral ☐
Beira Baixa ☐
Extremadura ☐
Ribatejo ☐
Alto Alentejo ☐
Baixo Alentejo ☐
Algarve ☐
Madeira ☐
Açores ☐

A9. **O alojamento está concebido para que animais?**

	Sim	Não
Cães	☐	☐
Gatos	☐	☐
Outras espécies	☐	☐

A10. **Qual o número atual (aproximadamente) de cães?**

A11. **Qual o número atual (aproximadamente) de gatos?**

Secção H: VIII. COMPORTAMENTO ANIMAL

H10. Onde são assistidos os animais, excluindo esterilizações?

Na instalação, pelo médico veterinário responsável ☐
Num Centro de Atendimento Médico Veterinário (CAMV) ☐
Na instalação, por médico veterinário externo ☐
Outro ☐

Secção I: IX. PERMANÊNCIA E ADOÇÃO

I1. **Qual a percentagem aproximada de animais que está no Alojamento há mais de 1 ano?**

	< 5%	5-10%	> 10-25%	> 25-50%	> 50%	Não aplicável
Cães	☐	☐	☐	☐	☐	☐
Gatos	☐	☐	☐	☐	☐	☐

Secção J: X. ASPECTOS CLÍNICOS

J1. Existem registos médicos individuais?

- Sim ☐
- Não ☐

J2. Quais as intervenções profiláticas realizadas por rotina?

- Desparasitação externa ☐
- Desparasitação interna ☐
- Vacinação ☐

J3. Existem restrições orçamentais para aplicação regular destas intervenções profiláticas?

- Sim ☐
- Não ☐

J4. **Quais as vacinas disponíveis para cães?**

- Raiva ☐
- Pentavalente DHPPi+L ☐
- Tosse Canil ☐
- Não há vacinas disponíveis ☐
- Não aplicável ☐

J5. **Quais as vacinas disponíveis para gatos?**

- Trivalente (RCP) ☐
- FeLV ☐
- Raiva ☐
- Não há vacinas disponíveis ☐
- Não aplicável ☐

J6. **Neste momento, qual é a cobertura vacinal dos animais alojados?**

	>80%	50-80 %	<50%	Não aplicável
Cães	☐	☐	☐	☐
Gatos	☐	☐	☐	☐

J7. **Quanto ao protocolo vacinal de cachorros e gatinhos, indique:**

- São vacinados repetidamente a cada 2-4 semanas? ☐
- O último reforço da primovacinação é dado após às 16 semanas de idade? ☐
- Outro ☐

Outro

J8. **Quanto ao protocolo vacinal de adultos, qual a periodicidade de revacinação?**

	Anual	De 3 em 3 anos	Nenhuma revacinação	Não aplicável
DHPPi+L nos cães	☐	☐	☐	☐
RCP nos gatos	☐	☐	☐	☐

J9. **Nos cães, qual é a ocorrência das seguintes doenças infecciosas nos últimos 12 meses?**

Neste inquérito considera-se "surto" como sendo 2 ou mais animais afetados no mesmo periodo.

	Nenhuma	1-2 casos (animais individuais)	>2 casos	Pelo menos 1 surto	Desconhecido	Não aplicável
Esgana	☐	☐	☐	☐	☐	☐
Parvovirose	☐	☐	☐	☐	☐	☐
Coronavirus canino	☐	☐	☐	☐	☐	☐
Tosse do canil	☐	☐	☐	☐	☐	☐
Sarna	☐	☐	☐	☐	☐	☐
Dermatofitose	☐	☐	☐	☐	☐	☐

J10. **Quais os principais métodos de diagnóstico?**

	Diagnóstico clínico	Teste rápido/ laboratorial	Necrópsia	Não aplicável
Esgana	☐	☐	☐	☐
Parvovirose	☐	☐	☐	☐
Coronavirus canino	☐	☐	☐	☐
Tosse do canil	☐	☐	☐	☐

J11. **Nos gatos, qual é a ocorrência das seguintes doenças infecciosas nos últimos 12 meses?**

Neste inquérito considera-se "surto" como sendo 2 ou mais animais afetados no mesmo periodo.

	Nenhuma	1-2 casos (animais individuais)	>2 casos	Pelo menos 1 surto	Desconhecido	Não aplicável
Panleucopenia felina	☐	☐	☐	☐	☐	☐
Coriza	☐	☐	☐	☐	☐	☐
Peritonite infecciosa felina	☐	☐	☐	☐	☐	☐
Sarna	☐	☐	☐	☐	☐	☐
Dermatofitose	☐	☐	☐	☐	☐	☐

J12. **Quais os principais métodos de diagnóstico?**

	Diagnóstico clínico	Teste rápido/ laboratorial	Necrópsia	Não aplicável
Panleucopenia felina	☐	☐	☐	☐
Coriza	☐	☐	☐	☐
Peritonite infecciosa felina	☐	☐	☐	☐

J13. **Nos gatos são realizados testes rápidos para retrovirus?**

- FIV ☐
- FeLV ☐
- Não são realizados ☐
- Não aplicável ☐

J14. **Nos gatos, um resultado positivo de teste rápido é confirmado por outro teste rápido ou laboratorial?**

- FIV ☐
- FeLV ☐
- Não há confirmação ☐
- Não aplicável ☐
